# Revisiting the usefulness of the short acute octreotide test to predict treatment outcomes in acromegaly

**DOI:** 10.3389/fendo.2023.1269787

**Published:** 2023-10-31

**Authors:** Montserrat Marques-Pamies, Joan Gil, Elena Valassi, Marta Hernández, Betina Biagetti, Olga Giménez-Palop, Silvia Martínez, Cristina Carrato, Laura Pons, Rocío Villar-Taibo, Marta Araujo-Castro, Concepción Blanco, Inmaculada Simón, Andreu Simó-Servat, Gemma Xifra, Federico Vázquez, Isabel Pavón, Rogelio García-Centeno, Roxana Zavala, Felicia Alexandra Hanzu, Mireia Mora, Anna Aulinas, Nuria Vilarrasa, Soledad Librizzi, María Calatayud, Paz de Miguel, Cristina Alvarez-Escola, Antonio Picó, Miguel Sampedro, Isabel Salinas, Carmen Fajardo-Montañana, Rosa Cámara, Ignacio Bernabéu, Mireia Jordà, Susan M. Webb, Mónica Marazuela, Manel Puig-Domingo

**Affiliations:** ^1^ Department of Endocrinology and Nutrition, Hospital Municipal de Badalona, Badalona, Spain; ^2^ Endocrine Research Unit, Germans Trias i Pujol Research Institute (IGTP), Badalona, Spain; ^3^ Centro de Investigación Biomédica en Red de Enfermedades Raras (CIBER-ER, Unidad 747), Instituto de Salud Carlos III (ISCIII), Barcelona, Spain; ^4^ Department of Endocrinology and Nutrition, Germans Trias i Pujol University Hospital, Badalona, Spain; ^5^ Department of Endocrinology and Nutrition, Arnau de Vilanova University Hospital, Lleida, Spain; ^6^ Endocrine Research Unit, Lleida Institute for Biomedical Research Dr. Pifarré Foundation (IRBLleida), Lleida, Spain; ^7^ Department of Endocrinology and Nutrition, Vall Hebron University Hospital, Barcelona, Spain; ^8^ Department of Endocrinology and Nutrition, Parc Taulí University Hospital, Sabadell, Spain; ^9^ Department Hormonal Laboratory, Germans Trias i Pujol University Hospital, Badalona, Spain; ^10^ Department of Pathology, Germans Trias i Pujol University Hospital, Badalona, Spain; ^11^ Department of Endocrinology and Nutrition, Clínico de Santiago University Hospital, Santiago de Compostela, Spain; ^12^ Department of Endocrinology and Nutrition, Ramón y Cajal University Hospital, Madrid, Spain; ^13^ Department of Endocrinology and Nutrition, Príncipe de Asturias University Hospital, Madrid, Spain; ^14^ Department of Endocrinology and Nutrition, Joan XXIII University Hospital, Tarragona, Spain; ^15^ Department of Endocrinology and Nutrition, Mutua de Terrassa University Hospital, Terrassa, Spain; ^16^ Department of Endocrinology and Nutrition, Josep Trueta University Hospital, Girona, Spain; ^17^ Department of Endocrinology and Nutrition, Getafe University Hospital, Madrid, Spain; ^18^ Department of Endocrinology and Nutrition, Gregorio Marañón University Hospital, Madrid, Spain; ^19^ Department of Endocrinology and Nutrition, Hospital Clinic University Hospital, Barcelona, Spain; ^20^ Endocrine Research Unit, Institut d’Investigacions Biomèdiques August Pi I Sunyer (IDIBAPS), Barcelona, Spain; ^21^ Department of Endocrinology and Nutrition, Hospital Sant Pau, Barcelona, Spain; ^22^ Universitat Autònoma de Barcelona (UAB), Departament de Medicina, Barcelona, Spain; ^23^ Department of Endocrinology and Nutrition, Bellvitge University Hospital, Bellvitge, Spain; ^24^ Endocrine Research Unit, Institut d’Investigació Biomèdica de Bellvitge (IDIBELL), Bellvitge, Spain; ^25^ Centro de Investigación Biomédica en Red de Diabetes y Enfermedades Metabólicas (CIBERDEM), Instituto de Salud Carlos III (ISCIII), Madrid, Spain; ^26^ Department of Endocrinology and Nutrition, 12 de Octubre University Hospital, Madrid, Spain; ^27^ Department of Endocrinology and Nutrition, Clínico San Carlos University Hospital, Madrid, Spain; ^28^ Department of Endocrinology and Nutrition, La Paz University Hospital, Madrid, Spain; ^29^ Department of Endocrinology and Nutrition, General University Hospital Dr Balmis, Miguel Hernández University, Alicante, Spain; ^30^ Endocrine Research Unit, Instituto de Investigación Sanitaria y Biomédica de Alicante (ISABIAL), Alicante, Spain; ^31^ Department of Endocrinology and Nutrition, La Princesa University Hospital, Madrid, Spain; ^32^ Department of Endocrinology and Nutrition, La Ribera University Hospital, Valencia, Spain; ^33^ Department of Endocrinology and Nutrition, La Fe University Hospital, Valencia, Spain

**Keywords:** acromegaly, somatostatin analogs, prediction, individualized treatment, precision medicine, acute octreotide test

## Abstract

**Introduction:**

We previously described that a short version of the acute octreotide test (sAOT) can predict the response to first-generation somatostatin receptor ligands (SRLs) in patients with acromegaly. We have prospectively reassessed the sAOT in patients from the ACROFAST study using current ultra-sensitive GH assays. We also studied the correlation of sAOT with tumor expression of E-cadherin and somatostatin receptor 2 (SSTR2) .

**Methods:**

A total of 47 patients treated with SRLs for 6 months were evaluated with the sAOT at diagnosis and correlated with SRLs’ response. Those patients whose IGF1 decreased to <3SDS from normal value were considered *responders* and those whose IGF1 was ≥3SDS, were considered *non-responders*. The 2 hours GH value (GH_2h_) after s.c. administration of 100 mcg of octreotide was used to define predictive cutoffs. E-cadherin and SSTR2 immunostaining in somatotropinoma tissue were investigated in 24/47 and 18/47 patients, respectively.

**Results:**

In all, 30 patients were responders and 17 were non-responders. GH_2h_ was 0.68 (0.25-1.98) ng/mL in responders vs 2.35 (1.59-9.37) ng/mL in non-responders (p<0.001). GH_2h_ = 1.4ng/mL showed the highest ability to identify responders (accuracy of 81%, sensitivity of 73.3%, and specificity of 94.1%). GH_2h_ = 4.3ng/mL was the best cutoff for non-response prediction (accuracy of 74%, sensitivity of 35.3%, and specificity of 96.7%). Patients with E-cadherin-positive tumors showed a lower GH_2h_ than those with E-cadherin-negative tumors [0.9 (0.3-2.1) vs 3.3 (1.5-12.1) ng/mL; p<0.01], and patients with positive E-cadherin presented a higher score of SSTR2 (7.5 ± 4.2 vs 3.3 ± 2.1; p=0.01).

**Conclusion:**

The sAOT is a good predictor tool for assessing response to SRLs and correlates with tumor E-cadherin and SSTR2 expression. Thus, it can be useful in clinical practice for therapeutic decision-making in patients with acromegaly.

## 1 Introduction

Somatostatin receptor ligands (SRLs) are the first-line medical treatment of patients with acromegaly (1–5). However, their efficacy is approximately 50% and treatment response assessment requires about 6 months (6–11). Furthermore, there have been more medical therapy options for acromegaly, and personalized medicine will be the focus of all treatment decisions in the near future. Then, it is of utmost importance to investigate predictive factors of the individual response for each patient (12–14).

As originally described, the acute octreotide test (AOT) is a functional test that consists of the administration of 100mcg of subcutaneous octreotide and the determination of the GH nadir or the decrease of GH during the following 6 hours. Its results have been related to long-term SRL response, and its utility has been extensively evaluated with some controversial results probably related to methodological differences: the use of 100 or 50mcg of octreotide for the test, the definition of the long-term response according to different parameters (GH levels, GH decrease, and IGF1 levels) and the use of different SRL presentations for long-term treatment (15–28). Moreover, the long duration of the originally described procedure limits its usefulness in clinical practice (4). In 2016, Wang et al. (15) reassessed the AOT predictive capacity with a very stringent methodology, obtaining remarkably good sensitivity and specificity values, but the methodology was still complex, time-consuming, and expensive. In 2008, we described a short version (sAOT) of the classic AOT where 100mcg of subcutaneous octreotide was administered, and the GH was determined at 2 hours post-administration (GH_2h_), as we found that this time point concentrated most if not all the nadir values. With this testing modality, we were able to predict the long-term response (more than 6 months) to SRLs with the cutoff for GH_2h_ of 3.6 ng/mL, which presented a negative predictive value (NPV) of 89% to identify non-response. This cutoff would help clinicians rule out the use of SRLs in monotherapy as initial medical therapy, and introduce pasireotide or pegvisomant earlier, alone or in combination with SRLs, thus shortening the time to control (18).

AOT results have also been linked to some particular molecular specificities such as the expression of SSTR2 and E-Cadherin (19, 29), both highly related to good SRL response mostly in terms of normalization of IGF1 (30).

Employing current GH ultra-sensitive assays, this study aimed to reevaluate prospectively the capacity of the sAOT to predict long-term response to SRLs in a cohort of patients with acromegaly included in the ACROFAST trial. We also aimed to evaluate the correlation of the functional predictive results of the sAOT with the expression of those molecular tumor biomarkers with high predictive capacity, such as E-cadherin and SSTR2.

## 2 Methods

### 2.1 Patients

Subjects from the cohort of the ACROFAST study were invited to participate in this sAOT substudy. ACROFAST is a prospective and multicenter trial that evaluates time to control in acromegaly using either a personalized or a standard sequential treatment approach and includes 21 tertiary referral centers in Spain. sAOT was performed prior to the initiation of treatment in all of the patients. None of the patients had received radiotherapy or any medical treatment for acromegaly in naïve cases and in those who failed to achieve remission after surgery, no treatment was started for a period of at least 3 months. In the ACROFAST study, two treatment modalities were evaluated: a standard treatment in which SRLs were given and a personalized modality in which SRLs, a combination with pegvisomant, or pegvisomant alone were considered according to the study protocol. However, sAOT was performed in all the patients of both treatment modalities, and in the experimental group, the sAOT result guided the treatment modality according to a pre-specified cutoff. Between December 2019 and June 2022, 47 patients who had been treated with SRLs in monotherapy for at least 6 months irrespective of the treatment modality were evaluated. SRLs were started at intermediate doses (Octreotide LAR 20mg or Lanreotide SR 90mg) and up-titrated to a maximal dose after 3 months if IGF1 was above the normal range. IGF1 was assessed at 3-month intervals in every patient. Of these 47 patients, 37 corresponded to newly diagnosed cases and 10 were non-cured surgical cases with significant remnant tumors.

The study was conducted in accordance with the ethical principles of the Declaration of Helsinki and implemented and reported in accordance with the International Conference on Harmonised Tripartite Guideline for Good Clinical Practice. The study was approved by the Germans Trias i Pujol Hospital Ethical Committee for Clinical Research (Ref.: PI-19-054). The protocol and informed consent forms were also approved by the institutional review board of all the participating centers, independent ethics committee, and/or research ethics board of each study site. All patients provided written informed consent to participate in the study.

### 2.2 Acute octreotide test

The sAOT consisted of collecting a basal blood sample for GH measurement, followed by the subcutaneous administration of 100 mcg of regular octreotide, and a second blood extraction 2 hours later. The GH_2h_ value was considered equivalent to the GH nadir (GH_nad_) as previously described (15). The percentage of GH fall from baseline (%ΔGH_2h_) was also used to evaluate GH suppression. Thereafter, all patients were treated with first-generation SRLs (Octreotide LAR or Lanreotide SR) administered monthly.

### 2.3 Response criteria

Patients were categorized according to the response based on the IGF1-Standard Deviation Scores (SDS) assessed after at least 6 months of SRLs treatment as 1) *Responders*, including complete responders (CR) when IGF1 was normalized (<2SDS) and partial responders (PR) when IGF1 was between 2 and 3SDS, and 2) *non-responders* (NR) when IGF1 was >3SDS over basal value (30, 31).

### 2.4 Magnetic resonance

Magnetic resonance imaging (MRI) was performed at baseline and between 3 and 6 months after the patients initiated treatment with SRLs to analyze changes in tumor size (highest diameter and volume) in either newly diagnosed cases or postsurgical ones. Tumor volume was calculated by the Di Chiro and Nelson formula: volume=height×length×width×π/6 (32). It was evaluated by an expert neuroradiologist in each center.

### 2.5 Immunohistochemistry

Formalin-fixed paraffin-embedded tumor samples were cut into sequential 4-µm-thick sections and stained using a fully automated Ventana BenchMark ULTRA stainer (Ventana, Tucson, AZ, USA) according to the manufacturer’s instructions.

E-cadherin immunohistochemistry (IHC) was performed in 24 tumors by using the mouse monoclonal anti-E-cadherin antibody (Ventana, Tucson, Ariz., USA) purchased as a prediluted antibody, with a concentration of 0.314 µg/dL. E-cadherin was scored in two intensities as previously described: negative [when the adenoma cells seemed negative at low and at high magnification (x40 and x200)] and positive [when the adenoma cells were positive at low (x40) or high magnification (×200)]. No differentiation was made between strong and weak positive E-cadherin adenomas because they have been described as showing the same response to SRL treatment (30).

SSTR2 IHC was performed in 18 tumors by using the rabbit monoclonal anti-SSTR2a antibody (clone UMB-1, Abcam) at a dilution of 1:100. Immunostaining for SSTR2 was scored by a semiquantitative immunoreactivity scoring system (IRS-Score). It was calculated by the product of the percentage of positive cells (0: no positive cells; 1: <10%; 2: 10–50%; 3: 51–80%; and 4: 80%) and the intensity of the staining (3, strong; 2, moderate; 1, mild; and 0, no staining), which resulted in IRS scores between 0 and 12 (33, 34). The cutoff of ≥5 was considered the limit to predict SRL response as described by Gatto et al. in 2013 (35).

The IHC studies were centralized in a single center and performed by an expert pathologist on pituitary tumors.

### 2.6 Hormonal determinations

Serum GH was measured at each center by different automated immunoassays, all calibrated against WHO IS 98/574: Immulite i2000, Siemens Healthineers (Erlangen, Germany) (23 patients), Liason XL, Diasorin (Saluggia, Italy) (17 patients), UniCel DxI 800 Access, Beckman Coulter (Brea, California) (4 patients), and Cobas 8000, Roche Diagnostics (Basel, Switzerland) (3 patients). Results were harmonized according to Müller et al. (36) with a linear regression equation for each assay that adjusted the GH concentrations of each immunoassay (x) to the results of the Immulite System (y). The Passing-Bablok regression equations were for Liason XL: y=1.272x + 0.023 and for DxI 800: y=1.387x + 0.356. To harmonize the results of the Roche assay we used the Passing-Bablok regression equation obtained by a method comparison of 51 samples measured by both immunoassays (Immulite i2000 and Cobas 8000). The regression equation obtained was y=1.089x + 0.082. All GH values presented in this research paper have been harmonized using this method.

Serum IGF1 concentrations were also measured in each center by immunoassays calibrated against WHO NISBC 2stIS 02/254: Liason XL, Diasorin (Saluggia, Italy) (39 patients), Immulite i2000 (Erlangen, Germany) (7 patients), and Elisa Mediagnost (Reutlingen, Germany) (1 patient). IGF1 was evaluated as absolute concentrations and as IGF1-SDS. IGF1-SDS were calculated using the calculator available online on the Spanish Society of Endocrinology and Nutrition website (www.seen.es/portal/calculadoras/sds-igf-1; last accessed 22 March 2023).

### 2.7 Statistical analysis

We calculated the statistical power of the study accepting an alpha risk of 0.05 and a beta risk of 0.2 in a two-sided test. We concluded that 16 subjects were enough in the non-responder (NR) group and 32 in the responder group (CR + PR) to recognize as statistically significant a difference greater than or equal to 1.75 ng/mL in the sAOT GH_2h_. The common standard deviation was assumed to be 2 for both groups.

Most continuous variables (GH_2h_, %ΔGH_2h_, basal and control GH, IGF1, and volume) showed a non-normal distribution evaluated by the Shapiro-Wilk test. Differences between means in responders and non-responders were assessed, respectively, with the Student t-test and Mann-Whitney U test or the Kruskall Wallis test when two or three different response groups were considered. Differences between categorical variables were assessed using Fisher’s exact test.

Correlations between numerical variables [age, Body Mass Index (BMI), height, GH_2h_, %ΔGH_2h_, basal and control GH, IGF1-SDS, tumor diameter and volume, IGF1 percentage variation (%ΔIGF1), tumor diameter and volume decrease, and SSTR2 IRS-score] were evaluated with Pearson’s and Spearman’s correlation coefficient.

A multivariate analysis was performed to exclude the presence of confounding factors between GH_2h_ or %ΔGH_2h_ and IGF1 response. Age, sex, BMI, height, basal GH, basal IGF1-SDS, basal tumor diameter, basal tumor volume, and GH assay were included as potential confounders.

Linear regression between GH_2h,_ %ΔGH_2h_, basal GH, basal IGF1, and IGF1-SDS after 6 months of SRL treatment was explored. The predictive values of the GH_2h_ and %ΔGH_2h_ during the sAOT to identify response to SRLs were appraised by binomial logistic regression receiver-operating characteristic (ROC) curves plotting sensitivity against 1-specificity. Basal GH and basal IGF1 were also explored with ROC curves.

A p-value of <0.05 was considered significant. Statistical analyses were performed using the R version 4.2.2 (R Project for Statistical Computing, RRID : SCR_001905). The graphical representation was done using package ggplot 2 (RRID : SCR_014601, Whickham https://CRAN.R-project.org/package=ggplot2) and the P values were added using ggpubr package (‘ggplot2’ Based Publication Ready Plots, https://CRAN.R-project.org/package=ggpubr). Finally, the ROC curves were plotted using the pROC package (Display and Analyze ROC Curves, https://CRAN.R-project.org/package=pROC).

## 3 Results

We analyzed a cohort of 47 patients with acromegaly (24 men and 23 women); the mean age at diagnosis was 53.6 ± 13.8 years. Of the total, 37 subjects corresponded to recently diagnosed patients and 10 to non-cured cases after surgery. A total of 30 out of 47 patients were identified as responders (26 CR and 4 PR) and 17 as NR. The group of 10 patients non-cured after surgery presented a slightly better response than the newly diagnosed patients (6 CR, 3 PR, and 1 NR, vs 19 CR, 1 RP, and 16 NR, respectively; p=0.01). Information and differences in clinical, hormonal, and radiological features between responders and NR patients are presented in **Table 1**. Responder patients were older and presented lower basal GH and IGF1 concentrations and lower tumor diameter. Apart from a better biochemical response, they presented a higher tumor volume shrinkage [Responders: 49.7 (12.2-89.7) % vs NR 12.5 (0-23.2) %; p=0.01].

**Table 1:** Basal cohort characteristics comparing responder and non-responder patients to SRLs after 6 months of medical treatment.

	RESPONDER (n=30)	NON-RESPONDER (n=17)	p
CLINICAL CHARACTERISTICS			
**Sex (♂/♀)**	15/15	9/8	1.00
**Age (Years)**	58 ± 13	47 ± 13	<0.01
**Weight (Kg)**	82 ± 17	86 ± 19	0.45
**Height (cm)**	170 ± 7	174 ± 11	0.20
**BMI (Kg/m^2^)**	28.8 ± 4.9	28.5 ± 4.8	0.84
**Hypertension** **(%; (n))**	46 (14)	11 (2)	0.02
**T2 Diabetes** **(%; (n))**	23 (7)	23 (4)	1.00
**Dyslipidemia** **(%; (n))**	37 (11)	29 (5)	0.75
**Sleep Apnea** **(%; (n))**	46 (14)	17 (3)	0.06
BASELINE BIOCHEMICAL AND TUMOR CHARACTERISTICS			
**GH (ng/mL)**	4.5 (2.7-9.2)	14.7 (7.1-24.8)	0.01
**IGF1 (ng/mL)**	489.5 (407.0-599.0)	760.0 (556.0-892.4)	<0.001
**IGF1 (SDS)**	4.7 (4.1-6.4)	8.1 (5.0-9.5)	<0.001
**Tumor Diameter (mm)**	13 ± 7	18 ± 9	0.05
**Tumor Volume (mm^3^)**	1051 (139-2163)	1876 (906-3990)	0.06

Insulin-like Growth Factor 1 (IGF1), Standard Deviation Scores (SDS), Body Mass Index (BMI), Type 2 Diabetes (T2 Diabetes), and Growth Hormone (GH).

### 3.1 sAOT results segregate SRL responders and non-responders

**Figure 1:**
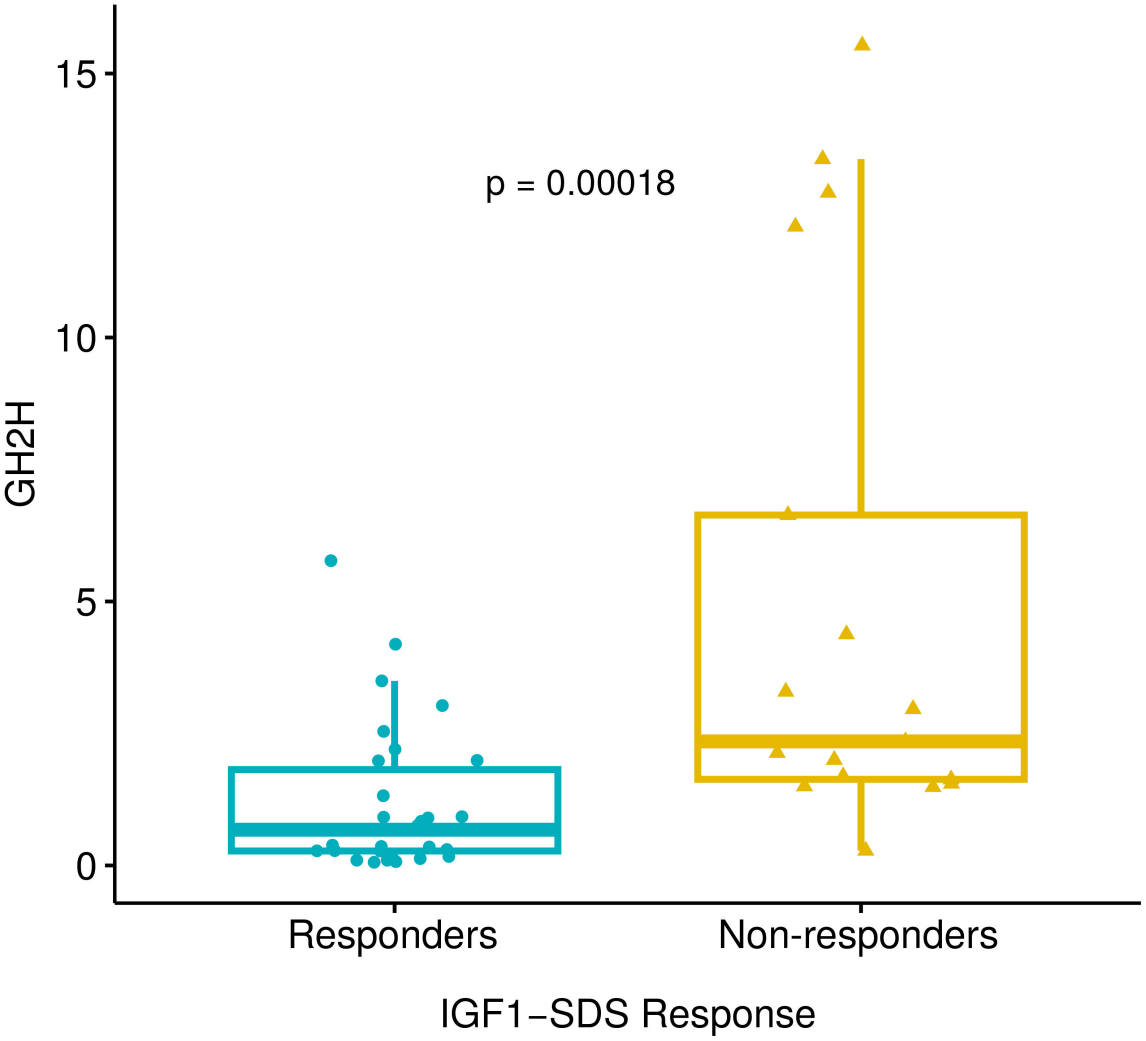
Differences in GH_2h_ between responder and non-responder patients analyzed with the Mann-Whitney U test. GH_2h_=0.68 (0.25-1.98) vs 2.35 (1.59-9.37) ng/mL; p<0.001. Responder patients include 26 complete responders (IGF1< 2SDS) and 4 partial responders (IGF1 2-3 SDS). There were 17 patients who were non-responders (IGF1>3SDS). Growth hormone at 2 hours after the short acute octreotide test (GH_2h_), Insulin-like Growth Factor 1 (IGF1), and Standard Deviation Scores (SDS).

sAOT GH_2h_ was significantly lower in responder patients as shown in **Figure 1**. When the three different treatment response categories were compared (CR, PR, and NR), the Kruskall Wallis test also showed significant differences between groups: GH_2h_=0.67 (0.15-1.65) (CR) vs 0.92 (0.29-4.98) (PR) vs 2.66 (1.61-8.00) (NR) ng/mL; p<0.001. GH_2h_ was also lower in patients with tumors <10mm [GH_2h_ 0.53 (0.28-1.52) vs 1.98 (0.52-3.16) ng/mL; p=0.02]. Even if GH_2h_ was lower in smaller tumors, the tumor size did not discriminate the response as there were no differences when the cutoff of 10 mm was considered for small and large tumors (CR=11 and NR=6 vs CR=14 and NR=11, respectively; p=0.75).

The %ΔGH_2h_ only showed a statistical trend, being higher in responder patients [87 (71–94) vs 79 (41–89) %; p=0.08]. Significant differences between extreme phenotypes (CR vs NR) were observed: 87 (81–95) vs 75 (42–89) %, respectively, p=0.02.

### 3.2 Correlation of the sAOT variables with basal analytical values and tumor size

**Figure 2:**
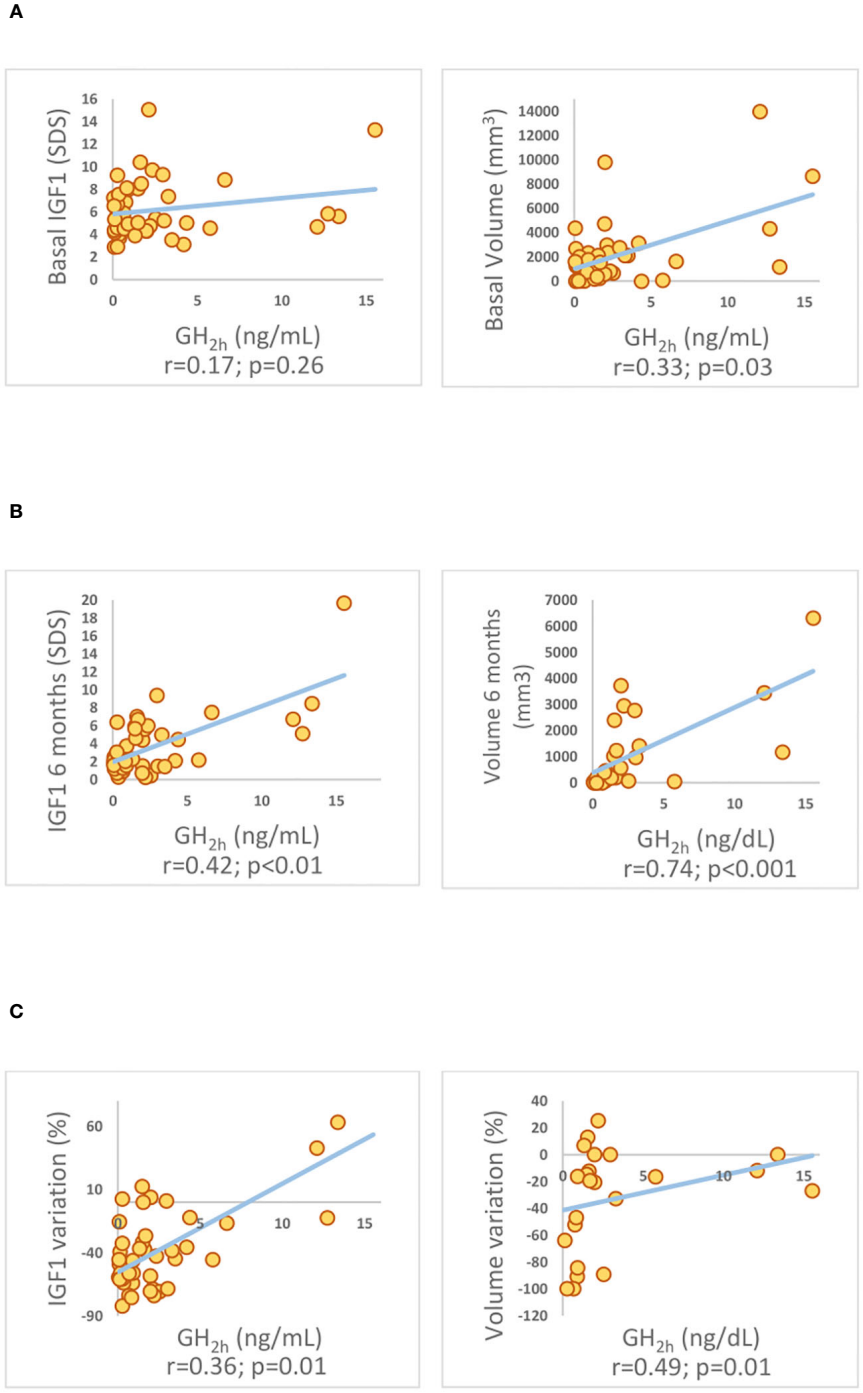
Spearman’s correlation coefficient among GH concentrations at 2 hours (GH_2h_) and biochemical parameters of control assessed by IGF1 concentration and tumor volume evaluated at 6 months of SRL treatment. **(A)** basal time-point, **(B)** after 6 months of medical treatment with SRLs, and **(C)** their variation over time **(C)**. Insulin-like Growth Factor 1 (IGF1). Standard Deviation Scores (SDS). IGF1 variation (%ΔIGF1).

GH_2h_ and %ΔGH_2h_ were positively correlated (r_s_ 0.51; p<0.01). GH_2h_ also showed a positive correlation with basal GH (r_s_ 0.60; p<0.001), maximal tumor diameter (r_s_ 0.37; p=0.01), and tumor volume (r_s_ 0.33; p=0.03), but not with baseline IGF1 (r_s_ 0.17; p=0.26). GH_2h_ correlated with all parameters of biochemical control at 6 months of follow-up: GH (r_s_ 0.51; p=0.001), IGF1-SDS (r_s_ 0.43; p<0.01), and tumor volume (r_s_ 0.74; p<0.001); and with the %ΔIGF1 (r_s_ 0.36; p=0.01), the ΔGH% (r_s_ 0.50; p<0.01), and the Δvolume% (r_s_ 0.49; p=0.02) (**Figure 2**). %ΔGH_2h_ only correlated with the %ΔIGF1 at 6 months (r_s_ 0.39; p<0.01).

The multivariate analysis excluded the presence of confounding factors in the association between GH_2h_ and the IGF1-SDS response (p<0.01). Moreover, basal IGF1 also showed a positive correlation with the IGF1 at 6 months (p<0.001).

### 3.3 Predictive value of sAOT to the response to SRLs

**Figure 3:**
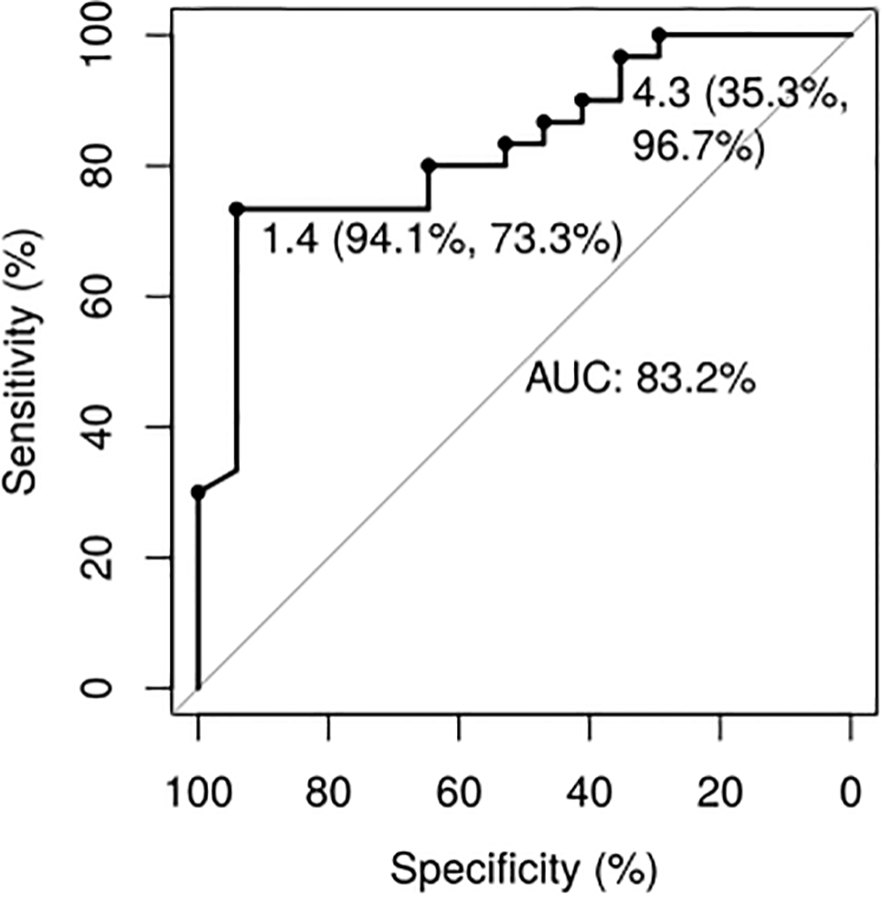
Growth hormone values 2 hours after the short acute octreotide test ROC Curves. The cutoff of 1.4ng/mL presented an accuracy of 81% for detecting responder patients (sensitivity of 73.3%, specificity of 94.1%, PPV of 96%, and NPV of 67%). The cutoff of 4.3ng/mL presented an accuracy of 74% for detecting non-responder patients (sensitivity of 96.7%, specificity of 35.3%, PPV of 86%, and NPV of 72%).

The binomial regression for treatment response showed an ROC curve for GH_2h_ with an Area Under the Curve (AUC) of 83.2% (p<0.001). Prognostic profiles for tumor response were established for two values of GH_2h_ (**Figure 3**). GH_2h_ = 1.4 ng/mL showed the highest power to identify responder patients with an accuracy of 81% [sensitivity of 73.3%, specificity of 94.1%, positive predictive value (PPV) of 96%, and NPV of 67%]. The cutoff value of GH_2h_=4.3ng/mL predicted non-response with an accuracy of 74% (sensitivity of 35.3%, specificity of 96.7%, PPV of 86%, and NPV of 72%) (**Figure 3**). The mean GH_2h_ for partial responders was 2.6ng/mL.

ROC curve of %ΔGH_2h_ indicated a non-predictive ability for SRLs response (AUC 65.5%; p=0.23).

### 3.4 Response to the sAOT is associated with tumor expression of E-cadherin and SSTR2

**Figure 4:**
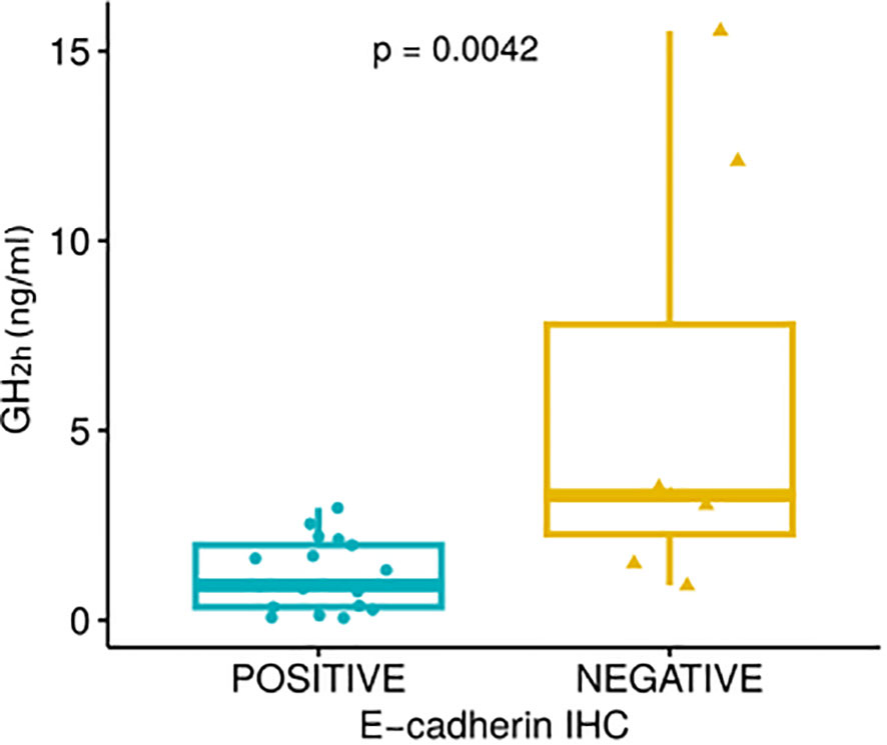
Growth hormone values 2 hours after the short acute octreotide test (GH_2h_) according to the positive or negative E-cadherin expression [GH_2h_ = 0.9 (0.3-2.1) ng/mL vs 3.3 (1.5-12.1) ng/mL; p<0.01] evaluated through the Mann-Whitney U test.

**Figure 5:**
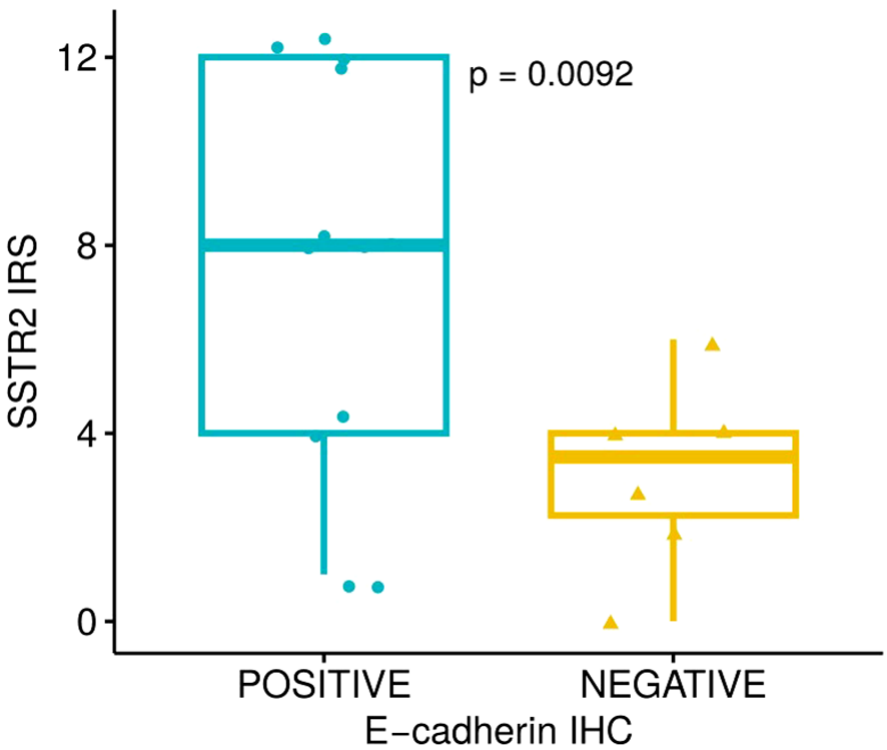
Somatostatin Receptor 2 (SSTR2) Immunoreactive Score (IRS-score) differences between tumors with positive and negative E-cadherin expression (3.3 ± 2.0 vs 7.5 ± 4.2; p=0.01) evaluated through Student-t test.

In those patients with available remnant tumor samples, we explored the association between tumor immunostaining for E-cadherin and SSTR2 with sAOT results: 24 and 18 patients, respectively (15 responders and 9 non-responders for E-cadherin and 12 responders and 6 non-responders for SSTR2). Lower GH_2h_ values were observed in E-cadherin positive cases: GH_2h_ = 0.9 (0.3-2.1) ng/mL vs 3.3 (1.5-12.1) ng/mL; p<0.01 (**Figure 4**). There were no differences in GH_2h_ according to the SSTR2 IRS-score described by Gatto (31) [GH_2h_ = 0.9 (0.2-1.9) ng/mL vs 2.2 (0.6-7.8) ng/mL; p=0.16] but patients with positive E-cadherin did present a higher SSTR2 expression (7.5 ± 4.2 vs 3.3 ± 2.0; p=0.01) (**Figure 5**).

### 3.5 Predictive value of basal GH and IGF1

Basal GH was lower in responders than in non-responders [4.5 (2.7-9.2) ng/mL vs 14.7 (7.1-24.8) ng/mL; p=0.01]. Basal GH correlated with both post-treatment GH (r_s_ 0.50; p<0.01) and post-treatment IGF1 (r_s_ 0.40; p<0.01). The ROC curve revealed an AUC of 77.1% for predicting the response (p=0.001), and basal GH of 10.5ng/mL was selected as the best predictor of non-response (accuracy of 77%, sensitivity of 64.7%, specificity of 83.3%, PPV of 68%, and NPV of 81%). It was not possible to identify a cutoff point that was accurate enough to identify responder patients.

Basal IGF1 was also lower in responders [4.7 (4.1-6.4) SDS vs 8.1 (5.0-9.5) SDS; p<0.001]. Expressed as %ULN: 205 (152–244) %ULN in responders and 319 (251 - 374) %ULN in non-responders. It correlated only with post-treatment IGF1 (r_s_=0.48; p<0.001). The ROC curve constructed showed an AUC of 80.5%; p<0.001. We obtained two relevant IGF1 cutoffs for defining response: 4.6 SDS with an accuracy of 67% (sensitivity of 50.0% and specificity of 94.1%) for responders and 8.0 SDS with an accuracy of 77% (sensitivity of 96.4% and specificity of 52.9%) for non-responders.

### 3.6 Newly diagnosed versus surgically non-cured patients’ response to sAOT

We performed the statistical analysis excluding the patients non-cured after surgery with the 37 newly diagnosed patients with acromegaly in order to avoid a biased selection and we obtained the same results. Basal characteristics (age, GH, IGF1, diameter, and volume) were different in responder patients compared with non-responder patients. GH_2h_ was lower in responders [GH_2h_=0.76 (0.28-1.98) ng/mL vs 2.24 (1.57-6.07) ng/mL; p=0.02]. The ROC curve presented an AUC for GH_2h_ of 78% with the highest accuracy for identifying response of 81% when GH_2h_ = 1.4 ng/mL (specificity of 88.2% and sensitivity of 70%). GH_2h_ was lower among patients with positive E-cadherin [GH_2h_=1.48 (0.66-2.15) ng/mL vs 3.16 (1.90-12.47) ng/mL; p=0.03], and SSTR2 expression was higher in those patients with positive E-cadherin expression (2.33 ± 2.08 vs 8.0 ± 3.13, p=0.02). Moreover, in the non-cured after surgical treatment group (n=10), remnant tumor volume was not different from tumor volume of new cases [volume = 756 (78-3336) mm^3^ vs 816 (165-2324) mm^3^; p=0.95], and the median GH_2h_ was neither different from the newly diagnosed cases [GH_2h_ = 0.36 (0.16-3.67) ng/mL vs 1.55 (0.53-2.75) ng/mL; p=0.26].

## 4 Discussion

Prediction of therapeutic response to a given compound with high accuracy for an individual patient is the cornerstone of precision medicine. Functional tests have been extensively used for diagnosis, evaluation of disease activity, and assessment of cure in most endocrine diseases. To be useful in clinical practice, a predictive test must be highly accurate, but it must also be easily implementable in the day-to-day clinical practice, and, if possible, inexpensive. The prediction of response to SRLs started historically in the early 90s when regular octreotide was given two to four times per day for controlling GH hypersecretion in patients with acromegaly (28). The GH_nad_ was used to schedule the number of doses to be delivered per day in each case by evaluating the GH drop after delivery of 100 mcg of regular octreotide in the subsequent hours. During the 1990s and 2000s, different authors evaluated the ability of the original 6 hours AOT - with sample collection every 60 minutes - to predict GH control in patients treated with long-acting SRLs with variable results and conclusions. Wang et al. reported in 2016 that this classic version of the AOT has a consistent capacity for prediction of SRL response in acromegaly (15), but it has never been included in the recommendations of clinical guidelines. We have previously reported that a short version of the classic AOT, in which GH_nad_ was established at 2 hours after octreotide injection, was reasonably predictive of the long-term response to SRLs (18). In that study, we found that a GH_2h_ cutoff of 3.6ng/mL was a measure of non-response with an NPV of 89% and that GH_2h_ presented a correlation of 0.76 with 6 months IGF1-SDS.

In the present study, we aimed to reevaluate our previously described cutoff points and their capacity to predict the response to SRLs by using a prospective cohort and current GH immunoassays. Our results confirmed the robustness of the AOT in this shorter version, with an accuracy of 81% (PPV for response 96%) and 74% (PPV for non-response 86%) for a cutoff of 1.4ng/mL and 4.3ng/mL, respectively. We also examined if tumor shrinkage was also predictable in parallel to biochemical response and we found that those cases showing a poor hormonal response were also those in which less marked volume reduction were found. Our results thus confirm that non-responder patients present an extremely low shrinkage compared to hormonal-responsive patients. The accuracy values obtained ranged from 81% for responsiveness to 71% for non-responsiveness, depicting the high heterogeneity of somatotroph tumors, especially those NR cases. However, the predictive ability shown in the present study may be more than sufficient to apply the sAOT in clinical practice, or at least indicates that it is much better to use it than not use it. In addition, this short version is easy to perform and inexpensive, and the results can be obtained quickly enough to promptly decide on medical treatment.

As expected, in the present study the cutoff values obtained are lower than those described in our previous study, which was published more than 12 years ago. The current values were obtained according to new immunoassays available, which have higher sensitivities. They go in parallel with the new, much lower cutoff values established in the latest clinical guidelines for the definition of acromegaly control and acromegaly remission after surgical treatment [GH<0.14ng/mL (4, 37) and <0.4ng/mL (5, 38)].

Most previous studies regarding AOT demonstrated its usefulness (15–18, 21–26, 28). However, some had methodological limitations that could have biased their interpretation, including retrospective studies, a small number of subjects, patients treated with radiotherapy, cumbersome octreotide presentations that could interfere with the adherence, and different response criteria (GH < 5mU/L, IGF1 normalization, IGF1 50% decrease or IGF1-SDS, among others). One of the relevant findings of the present study is the robust correlations observed, as in other studies, between the GH decrease observed at AOT and the long-term GH decrease (16, 21); these decreases were observed between GH_2h_ and basal GH (19, 22), and between post-treatment GH (17, 22, 28) and post-treatment IGF1 (17, 18), with mixed results regarding GH_2h_ and basal IGF1 presenting a positive correlation in some studies (17, 22) but not in others (18, 21). A different GH_nad_ between responders and non-responders has also been previously described (15, 17, 23), which is in fact, the key to its application in clinical practice. Our correlation analysis and the differences found in GH_2h_ and the %ΔGH_2h_ among responder and non-responder patients are concordant with the results described in these studies.

We also investigated the ability of basal GH and IGF1 to predict SRL response, as previously described by other researchers (17–19, 21, 39–41). The predictive ability of both parameters can be useful, but only IGF1 is able to define the cutoff point for responders and non-responders. Moreover, our data show that GH_2h_ is the best predictor tool, being able to identify both responders and non-responders, showing the best accuracy and predictive values, and being consistent enough for use in clinical practice.

Patients with smaller tumors presented a lower GH_2h_ and a better response as reported by other studies (16). Wang et al. described an AUC of 0.794 for the AOT ability to predict tumor volume reduction (15). In the present study and concordant with these previous findings, GH_2h_ also correlated with volume decrease.

Non-cured after-surgery patients presented better responses to SRL treatment than the newly diagnosed ones, probably due to the debulking surgery effect as previously described (42, 43). However, we did not observe in our cohort a lower tumor volume in this group prior to sAOT and treatment initiation. GH_2h_ in post-surgical active disease cases did not show statistical differences compared with newly diagnosed patients, although the results could be influenced by the small number of non-cured after-surgery patients. When we performed the statistical analysis focused only on newly diagnosed patients, we obtained the same results as when pooling all the cases. This data reinforces that sAOT can be useful either in post-surgery patients or in newly diagnosed cases.

Interestingly, GH_2h_ was lower among positive E-cadherin tumors. Patients with positive E-cadherin did present a higher SSTR2 expression confirming that both biomarkers are usually expressed concordantly (30, 44). Casar-Borota described an association between SSTR2 IRS-score and AOT results (r_s_ -0.29; p=0.02) (45), which was not replicated in our study. Moreover, the differences in GH_2h_ obtained according to E-cadherin but not to SSTR2 immunostaining showed a higher predictivity of the former biomarker, as previously reported (30). When E-cadherin is present in GH-secreting tumors, it seems to reflect a greater phenotypic somatotropic differentiation (46–48), confirming that it is a comprehensive molecular marker not just reflecting intercellular adhesion, but also including the information regarding the responsiveness to SRL treatment (30, 49, 50). The IHC determination of E-cadherin is a cheaper, easily evaluable, robust, and daily-used technique in all pathology clinical laboratories, which can facilitate its implementation for somatotropinomas theragnostic assessment; and, interestingly, with no inferior (or even superior) properties to predict SRL response compared with SSTR2 (30). For these reasons, we consider that E-cadherin should be implemented in clinical practice even before SSTR2 immunostaining.

The present work has some strengths: it is prospective, and the number of patients is sufficient for a proof-of-concept study. One limitation to defining the GH_2h_ cutoff is the use of different GH assays (51). To overcome this aspect, we have harmonized GH values as Müller et al. described (36). They made comparisons between GH determinations obtained from children and adolescents with suspected GH deficiency through eight different assays, three of which were used in the present study (Immulite i2000, Liaison XL, and UniCel DxI 800 Access). Assay standardization and different statistical harmonization strategies were performed. Using the linear regression equations, inter-laboratory assay variability was reduced from a CV of 68.21 ± 45.6% to 32.3% ± 29.0% for GH <1 ng/mL and from 28.2% ± 11% to 15.4% ± 11.7% for GH 1-4.99 ng/mL, thus, enhancing the possibility to use our GH cutoffs with all the GH assays included in the present work. On the other hand, some inconsistencies or relatively unusual clinical situations must be clarified, such as those subjects with a basal GH value lower than the theoretical cutoff point for responsiveness. We identified three cases with a basal GH <1.4 ng/mL. Of these three, two were included after surgery and presented a %ΔGH_2h_ of 89% and 76%. Both presented a complete response to SRLs. The third case was a newly diagnosed acromegalic patient with a %ΔGH_2h_ of 19% who did not respond to SRLs. This information exemplifies that even if basal GH is relatively low, the sAOT retains a good capacity to predict long-term response to SRLs and to assess a predictive response to medical treatment. In addition, the use of different assays for measuring IGF1 may limit the interpretation of our results. Even if the use of SDS or %ULN may allow comparability among centers using different IGF1 assays, the upper limit of normality of the different assays we used shows relatively different values (52), thus, the final upper cutoff from assays to define responsiveness may also be affected.

Currently, SRLs are the established first-line medical treatment for all patients. Tools such as sAOT can help clinicians overcome therapeutic ineffectiveness when a GH_2h_ is high and a non-response to SRLs is expected, which can be quickly confirmed with this functional test. Thus, in those cases with a prediction of non-response, the primary use of SRLs could be avoided and the use of pasireotide or pegvisomant as first-line medical treatment would be recommended according to the specific characteristics of the patient and the tumor. Time and cost would be potentially saved with such an approach. In conclusion, we report that the short version of the AOT performed within 2 hours from the time of diagnosis or soon after surgical failure, and using the GH immunoassays included in the present paper, is robust enough for its implementation as a predictive SRL response tool at the clinical practice level and is capable of guiding the medical treatment of acromegaly.

## Data availability statement

The raw data supporting the conclusions of this article are available from the corresponding author on reasonable request.

## Ethics statement

The study was approved by the Comite d’ètica i investiació de l’Hospital Germans Trias i Pujol. The study was conducted in accordance with the local legislation and institutional requirements. The participants provided their written informed consent to participate in this study.

## Author contributions

MM-P: Formal Analysis, Methodology, Writing – original draft. JG: Formal Analysis, Supervision, Writing – review & editing. EV: Writing – review & editing. MH: Writing – review & editing. BB: Writing – review & editing. OG-P: Writing – review & editing. SM: Writing – review & editing. CC: Methodology, Writing – review & editing. LP: Methodology, Writing – review & editing. RV-T: Writing – review & editing. MA-C: Writing – review & editing. CB: Writing – review & editing. ISi: Writing – review & editing. AS: Writing – review & editing. GX: Writing – review & editing. FV: Writing – review & editing. IP: Writing – review & editing. RG-C: Writing – review & editing. RZ: Writing – review & editing. FH: Writing – review & editing. MMo: Writing – review & editing. AA: Writing – review & editing. NV: Writing – review & editing. SL: Writing – review & editing. MC: Writing – review & editing. PM: Writing – review & editing. CA-E: Writing – review & editing. AP: Writing – review & editing. MS: Writing – review & editing. ISa: Writing – review & editing. CF-M: Writing – review & editing. RC: Writing – review & editing. IB: Writing – review & editing. MJ: Writing – review & editing. SW: Writing – review & editing. MMa: Writing – review & editing. MP-D: Conceptualization, Funding acquisition, Supervision, Visualization, Writing – review & editing.
